# Fatal vanishing bile duct syndrome in Iranian patient with Hodgkin's lymphoma

**DOI:** 10.1002/ccr3.7671

**Published:** 2023-07-19

**Authors:** Fazlollah Shokri, Aref Shariati, Arash Kazemi Veisari, Amirhossein Kianezhad, Somayeh Sheidaei, Ali Asghar Alamian, Hossein Sadeghi, Mohsen Heidary

**Affiliations:** ^1^ Department of Medical Genetics, Faculty of Medical Sciences Tarbiat Modares University Tehran Iran; ^2^ Molecular and Medicine Research Center Khomein University of Medical Sciences Khomein Iran; ^3^ Department of Internal Medicine, Gut and Liver Research Center, School of Medicine Mazandaran University of Medical Sciences Sari Iran; ^4^ Faculty of Medicine Mazandaran University of Medical Sciences Sari Iran; ^5^ Department of Laboratory Sciences, Faculty of Paramedical Sciences Mazandaran University of Medical Sciences Sari Iran; ^6^ Department of Orthopedics, Faculty of Medicine Shahid Beheshti University of Medical Sciences Tehran Iran; ^7^ Genomic Research Center Shahid Beheshti University of Medical Sciences Tehran Iran; ^8^ Department of Laboratory Sciences, School of Paramedical Sciences Sabzevar University of Medical Sciences Sabzevar Iran; ^9^ Cellular and Molecular Research Center Sabzevar University of Medical Sciences Sabzevar Iran

**Keywords:** ductopenia, Hodgkin's lymphoma, liver failure, vanishing bile duct syndrome

## Abstract

Vanishing bile duct syndrome (VBDS) has been postulated that may be related to Hodgkin's lymphoma (HL). In the present study, we present a 75‐year‐old male patient with HL who received chemotherapy but has not received any radiotherapy. The patient's condition worsened in further days, and he died with the diagnosis of cirrhosis and hepatic failure.

## 1 INTRODUCTION

Vanishing bile duct syndrome (VBDS) is a group of genetic and acquired disorders that lead to the progressive disappearance and destruction of intrahepatic bile ducts (ductopenia).^1^, ^2^ Although the exact pathogenesis of VBDS is poorly understood, recent studies have reported Hodgkin's lymphoma (HL) as one of the probable causes of VBDS.^2^, ^3^

Hodgkin's lymphoma is a type of cancer that lymphocytes grow out of control, causing swollen lymph nodes. Hodgkin's lymphoma can also be seen in bone marrow transplant patients.^4^, ^5^

HL‐associated jaundice has been reported to occur in a low percentage of patients (3%–13%), while the involvement of hepatic is uncommon.^6^ Various causes, such as hepatic infiltration, biliary obstruction, or viral infections, could lead to jaundice in HL patients.^7^

Noteworthy, HL‐related VBDS is a rare condition that has been described in a number of cases and often leads to liver failure and death. Additionally, this disease is considered a paraneoplastic process that commonly presents with pruritus, jaundice, and weight loss.^3^, ^6^, ^7^ Most of the data about HL‐related VBDS are collected primarily from case reports; accordingly, there are limited data about the perfect treatment and clinical course for this disease.^3^, ^8^

Therefore, more clinical studies are required for a better understanding of the diagnosis and management of the HL‐related VBDS. To this end, we present a 75‐year‐old Iranian male patient with HL‐related VBDS that expired when he was under chemotherapy.

## 2 CASE PRESENTATION

A 75‐year‐old Iranian male known case of HL (mixed cellularity) who received previously chemotherapy, and has not received any radiotherapy was admitted to the hospital with fever, anorexia, icterus, and generalized pruritus. Furthermore, the patient reported dark urine (2 weeks), while stool color has not changed.

The patient was receiving chemotherapy for his HL and was in the remission phase. In physical examination, he was ill and cachectic with fever, generalized jaundice, and paleness. A surgical scar from a previous excisional biopsy of lymph nodes was seen, but lymphadenopathy was not detected, and chest, abdomen, and extremity examinations were normal. Laboratory findings are presented in Table 1. Due to the cholestatic pattern of liver enzymes, ultrasonography and magnetic resonance cholangiopancreatography (MRCP) were requested, and both of them were normal.

**TABLE 1:** Different laboratory findings in patients with HL‐related VBDS.

WBC	7.39 × 10^3^/μL (4.5–11 × 10^3^)	INR	2.1 → 1.3
Neutrophils	65% (45–75)	PTT	38 s (25–40)
Lymphocyte	18% (20–40)	PT	22.3 s (12–15.1)
Monocyte	14% (1–10)	CRP	2+
RBC	4.2 × 10^6^/μL (3.8–5.5)	ESR	64 mm/h (1–20)
HB	9.2 g/dL (12–16)	FBS	107 mg/dL (70–105)
MCH	21.7 pg (27–32)	Cholesterol	149 mg/dL (<200)
MCV	960.5 fL (80–95)	Triglycerides	342 mg/dL (<200)
Hematocrit	25.6% (35–47)	K	3.9 mEq/L (3.5–5.5)
M.C.H.C	35.9 g/dL (32–37)	NA	133 mEq/L (135–145)
RDW	23.6% (11–14.7)	Amylase	100 IU/L (up to 100)
PLT	288 × 103/mm^3^ (140–450)	Lipase	44 U/L (up to 38)
Urea	175 mg/dL (13–43)	Cr	2.09 → 6 mg/dL (0.6–1.4)
Urine analysis	Normal	Stool exam	Normal
Bilirubin total	35 mg/dL (<1.2)	Lactate dehydrogenase (LDH)	344 IU/L
Bilirubin direct	16 mg/dL (<0.3)	Gamma‐glutamyl transferase (GGT)	Normal
Alkaline phosphatase (ALP)	1839 U/L (80–306)	Serum albumin (Alb)	2.7
Aspartate aminotransferase (AST)	78 U/L (up to 37)	Total protein	6.1 g/dL (6.6–8.8)
Alanine aminotransferase (ALT)	55 U/L (0–41)		

**FIGURE 1:**
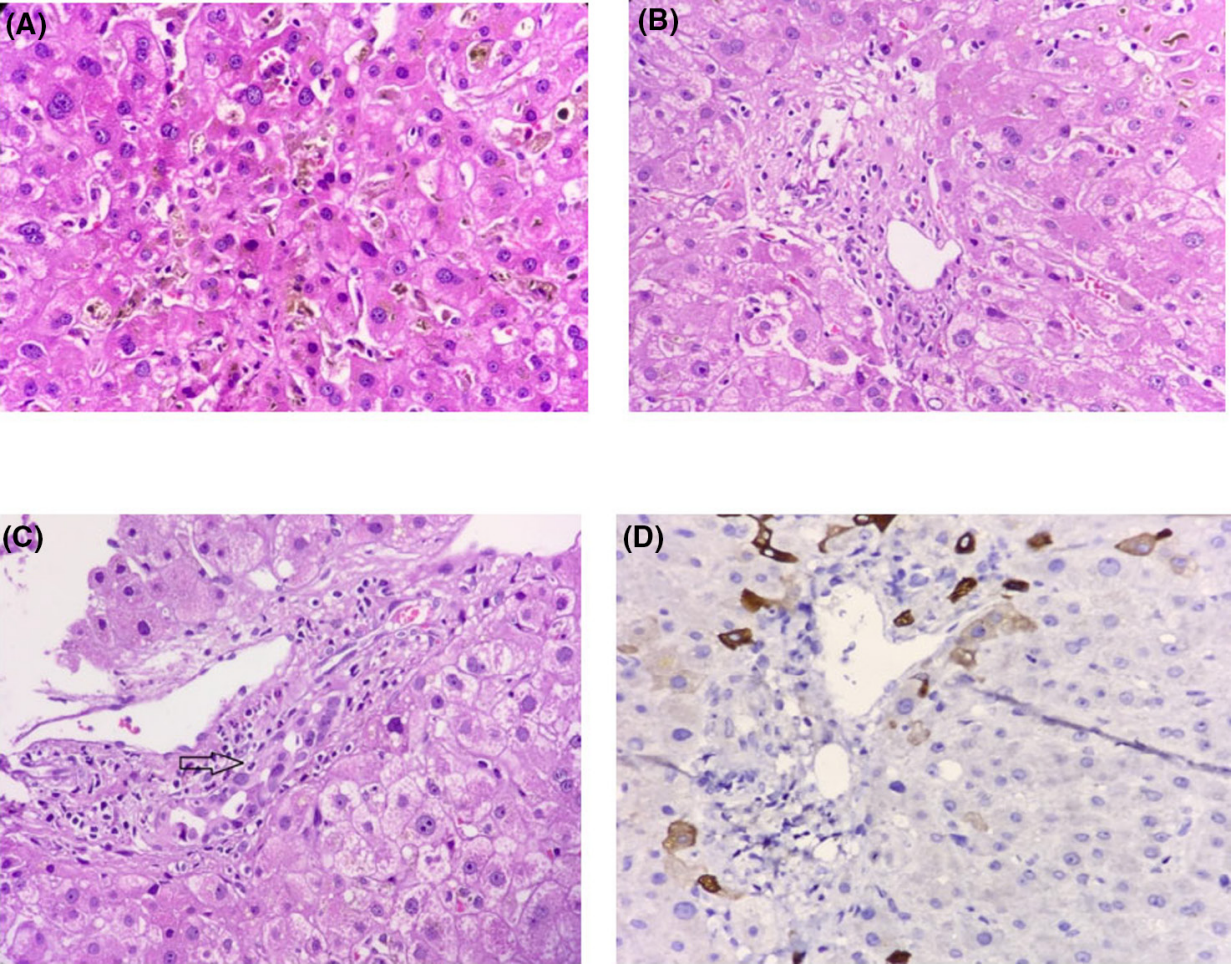
Pathologic findings in a patient with HL‐related VBDS. (A) Cholestasis with bile pigment deposition in hepatocytes. (B) Loss of bile duct in the portal tract. (C) The interlobular bile duct (black arrow) with vacuolated and irregular epithelium, as well as infiltrated by lymphocytes (H&E stain, 400× magnification). (D) Absence of bile duct in cytokeratin seven immunohistochemistry (IHC) staining (IHC staining 400×).

Afterward, relevant tests were requested, and all of them were in the normal range (Table 2). In the next step, a liver biopsy was done, which showed severe intrahepatic cholestasis with marked bile duct epithelial injury of more than 50% ductopenia. Additionally, severe hepatocellular and canalicular bilirubinostasis were detected, and the portal spaces showed marked bile duct injury with a decreased number, as well as moderate chronic inflammation. On the contrary, trichrome staining did not demonstrate any evidence of fibrosis. Collectively, the pathologic diagnosis was VBDS (Figure 1).

**TABLE 2:** Various screenings were done on a patient with HL‐related VBDS.

Autoimmune disorders screening and tumors markers	
LKM	2.4 AU/mL (<12)
Anti‐nuclear Ab	1/80 (pattern speckled) (<1/80)
Anti‐mitochondria lab	0.9 (<10)
Anti‐smooth muscle Ab	1/40 (<1/80)
Nuclear membrane pattern	Seen^a^
CA 19‐9	33.8 U/mL (up to 39)
CEA	0.8 ng/mL (0–4)
Alpha FP	Negative
RF	Negative
P‐ANCA	Negative
C‐ANCA	Negative
IgG4	36
**Virology analysis**	
CMV (IgM)	0.41 COI (<0.70)
EBV Ab (VCA) (IgM)	0.01 Index (≤0.11)
HAV Ab (IGM)	0.2 Index (<0.9)
HEV Ab (IGM)	Negative
**Serum protein electrophoresis**	
Alpha1 globulin	4.2% (2.9–4.9)
Alpha2 globulin	10.6% (7.1–11.8)
Beta1 globulin	4.7% (4.7–7.2)
Beta2 globulin	7.8% (3.2–6.5)
Gamma globulin	27.4% (11.1–18.8)
Albumin	45.3% (55.8–66.1)

^a^ Antibodies against nuclear membrane occur in Primary Biliary Cirrhosis (PBS).

In the next days, the patient's general conditions worsened, and he developed drowsiness and abdominal ascites; and diagnostic tap revealed high Serum Ascites Albumin Gradient (SAAG) ascites. Hydrocortisone 100 mg IV (TDS) and ursodeoxycholic acid (UDCA) Tablet 300 mg (BD) were started for the patient due to the previous pathologic report, inflammation, SPEP, and cholestatic pattern of liver enzymes. Additionally, antibiotics (metronidazole 250 mg Tablet (BD) and Ceftriaxone 1gr vial (infusion BD & Stat)) and vitamin K 1 mg (SC/Daily) were prescribed for patient fever of unknown origin and prolonged coagulation time, respectively.

During admission, the patient experienced an increase in creatinine from 2.1 to 6 mg/dL, and according to the nephrology consult, the patient got three dialysis sessions. Because of his past medical history, we consulted with an oncologist, but he did not accept chemotherapy due to the high level of bilirubin. Finally, despite performing all the mentioned treatments, the patient's condition worsened in further days, and he died with the symptoms of cirrhosis, hepatic and renal failure.

## 3 DISCUSSION

Ductopenia is defined as a decrease in the number of intrahepatic bile ducts, a disorder that could finally cause biliary cirrhosis and cholestatic liver disease. In this regard, VBDS refers to a cluster of disorders leading to the ductopenia and eventually, liver failure, cirrhosis, and cholestasis, as well as death.^9^ VBDS has a variable prognosis and is partially dependent on the bile duct injury etiology.^3^ The exact processes in this disease are not yet known; however, the available literature reported immune‐related pathogenesis as one of the most important etiological factors in VBDS.^10^

Additionally, VBDS has been reported in patients with HL, where it is thought to be a paraneoplastic phenomenon.^3^, ^8^ VBDS, secondary to HL, is a rare cause of cholestasis in these patients. The exact mechanism of VBDS in patients with HL is poorly reported; however, a paraneoplastic effect seems most likely.^11^

In this presented case, due to the pathologist's report and rule out of the autoimmune condition, as well as the lack of infiltration, HL was considered the probable cause of VBDS. The suitable management of HL‐related VBDS remains controversial, as both conventional chemotherapy and alternative regimens have been reported to be successful in achieving remission in this condition.^9^, ^12^, ^13^, ^14^ Additionally, radiotherapy could increase liver failure‐free survival, while chemoradiation is a treatment option that in many patients finally causes remarkable liver failure, needing liver transplantation.^12^, ^15^

The available literature has reported a high mortality rate in patients with HL‐related VBDS despite adequate treatment. Notably, this high mortality rate is mostly related to liver dysfunction rather than lymphoma progression. This clarifies the difficulties encountered in the administration of potential hepatotoxic chemotherapy in severely cholestatic patients.^8^, ^9^

In our patient, chemotherapy was rejected by an oncologist because of the high level of bilirubin. Accordingly, the patient had been treated conservatory but, unfortunately, died 4 weeks after admission with the diagnosis of cirrhosis, hepatic, and renal failure.

## 4 CONCLUSION

In this case report, a 75‐year‐old male patient with HL who received chemotherapy was described. The patient's condition worsened, and he died with the diagnosis of cirrhosis and hepatic failure. The exact HL‐related VBDS pathogenic mechanisms were not detected; however, it seems lymphoma‐induced toxic cytokine release directly damages the bile ducts. Furthermore, these cytokines also destroy the bile duct through occult infiltration and other effector cells recruitment.

## AUTHOR CONTRIBUTIONS

**Fazlollah Shokri:** Conceptualization. **Aref Shariati:** Data curation. **Arash Kazemi Veisari:** Data curation; methodology. **Amirhossein Kianezhad:** Validation. **Somayeh Sheidaei:** Writing – review and editing. **Ali Asghar Alamian:** Writing – original draft. **Hossein Sadeghi:** Writing – review and editing. **Mohsen Heidary:** Supervision.

## FUNDING INFORMATION

None of the authors received funding for writing this article.

## CONFLICT OF INTEREST STATEMENT

The authors declare that they have no conflict of interest.

## CONSENT

Written informed consent was obtained from the patient to publish this report in accordance with the journal's patient consent policy.

## Data Availability

Data sharing is not applicable to this article as no datasets were generated during the current study.
